# Various Surgical Uses of Vulcanized India-Rubber

**Published:** 1850-09

**Authors:** 


*Various Surgical Uses of Vulcanized India-Rubber.*—Vulcanized caoutchouc has now become very common in the arts, and is likewise used in surgery. A systematic and extensive application of this substance has lately been made at Paris, for surgical purposes; and Dr. GARIEL presented to the Surgical Society of that capital, on the 19th of September last, a series of apparatuses, made of vulcanized caoutchouc, for traction, compression, confinement, dilatation, suction, &c. Dr. Gariel prepares the substance by dipping the caoutchouc into a saline bath, and thereby renders the India-rubber perfectly and regularly elastic, gives to it an immense force of cohesion, prevents fatty bodies or the most energetical chemical agents (nitrate of silver, nitric, sulphuric, and muriatic acids, &c.,) from acting upon it, and causes it to preserve its elasticity at extreme temperatures. It should be remembered that ordinary caoutchouc possesses none of the above-named properties. One peculiarity pervades the numerous kinds of apparatuses which Dr. Gariel laid before the Society, and that is, a very ingeniously-contrived mode of insufflating various tubes, when placed upon, or introduced into, the body, giving them a greater diameter, and increasing their power of pressure at will. We may mention a tube used for arresting epistaxis, which will plug the posterior nares by insufflating it. Pessaries and a great number of variously shaped cushions, intended for many different surgical uses are also made to change their size at will, by filling them more or less with air. The Society expressed its highest approval of these applications of vulcanized caoutchouc.—*Lancet, Dec,* 1, 1848, *p.* 579.

